# Supplementary material for: Helicobacter pylori infection is associated with fecal biomarkers of environmental enteric dysfunction but not with the nutritional status of children living in Bangladesh
Source: PLoS Negl Trop Dis. 2020 Apr 23;14(4):e0008243. doi: 10.1371/journal.pntd.0008243 (PMC7200013; doi:10.1371/journal.pntd.0008243)
Supplement: S1 Table — (DOCX) [file pntd.0008243.s002.docx]

**S1 Table. Variables used in this analysis**

|  | **Variable** | **Types of variable** | **Definition** |
| --- | --- | --- | --- |
| Independent variable | *H. pylori* infection | Categorical | Stool specimens with absorbance values ≥0.15 were considered positive and specimens with absorbance values <0.15 were considered negative for infection with *H. pylori*. |
| Outcome variables | Alpha-1-antitrypsin (AAT) | Continuous | Biomarker of barrier dysfunction and enteric protein loss |
|  | Myeloperoxidase (MPO), Neopterin (NEO), and Calprotectin | Continuous | Markers of intestinal inflammation |
|  | Reg1B | Continuous | Marker of intestinal injury and repair |
|  | LAZ, WAZ, WLZ | Continuous | Indicators of nutritional status of children |
| Covariates | Treatment of drinking water | Categorical | Whether treat drinking water or not |
|  | Source of drinking water | Categorical | Sources of drinking waters |
|  | Source of cooking water | Categorical | Sources of cooking water |
|  | Hand washing practice after toilet | Categorical | Always wash their hands after toilet or not |
|  | Hand washing practice after child defecation | Categorical | Always wash their hands after helping the child to defecate or not |
|  | Hand washing practice before cooking | Categorical | Always wash their hands before cooking or not |
|  | Crowded living conditions | Categorical | More than 4 people sleep in a single room or not |
|  | Separate space for kitchen | Categorical | Having separate space for kitchen or not |
|  | Exposure to domestic animals | Categorical | Whether exposed to animals in households or not |
|  | Mother received education | Categorical | Mother received formal education or not |
|  | Household head received education | Categorical | Household head received formal education or not |
|  | CRP, AGP | Continuous | Markers of systemic inflammation |
