# Supplementary material for: Helicobacter pylori infection is associated with fecal biomarkers of environmental enteric dysfunction but not with the nutritional status of children living in Bangladesh
Source: PLoS Negl Trop Dis. 2020 Apr 23;14(4):e0008243. doi: 10.1371/journal.pntd.0008243 (PMC7200013; doi:10.1371/journal.pntd.0008243)
Supplement: Attachment — Submitted filename: Point-by-point responses.docx [file pntd.0008243.s003.docx]

**Responses to the review comments**

Dear Editor and Reviewers,

Thank you very much for reviewing the manuscript entitled "*Helicobacter pylori* infection is associated with fecal biomarkers of environmental enteric dysfunction but not with the nutritional status of children living in Bangladesh". Please find below the responses to the comments made by the reviewers and a description of the changes made in the manuscript.

Reviewer's Responses to Questions:

*<b>Key Review Criteria Required for Acceptance?</b></br></br>*

*As you describe the new analyses required for acceptance, please consider the following:</br></br>*

*<b>Methods</b></br></br>*

*-Are the objectives of the study clearly articulated with a clear testable hypothesis stated?</br>*

*-Is the study design appropriate to address the stated objectives?</br>*

*-Is the population clearly described and appropriate for the hypothesis being tested?</br>*

*-Is the sample size sufficient to ensure adequate power to address the hypothesis being tested?</br>*

*-Were correct statistical analysis used to support conclusions?</br>*

*-Are there concerns about ethical or regulatory requirements being met?</br></br>*

Reviewer #1: This is a carefully designed study with a robust study design undertaken as part of a much larger research programme into environmental enteropathy in Dhaka. The population is carefully described and appropriate although the lack of well-nourished controls is acknowledged by the authors as a potential limitation of the study. The sample size is more than adequate and the statistical analysis is thorough and clearly described. Appropriate ethical approvals were obtained for the study protocol.

**Response:** Thank you very much for your very positive comments on study design, sample size, statistical analyses, and ethical approvals.

Reviewer #2: Though the objectives are clearly mentioned and testable but the reason for selection of these objectives and study are not clearly mentioned. It is not mentioned why this study is needed and what will be the impact of results of this study. How finding any association or lack of association between H. pylori infection and fecal biomarkers in EED will advance any significant and useful scientific knowledge.

**Response:** *Helicobacter pylori* infection is highly prevalent in low-income countries and affects more than half of the global population (1). H. pylori is known to be associated with gastritis as well as protein losing enteropathy with evidence of resolution of the enteropathy by eradication of the infection (2, 3). Environmental Enteric Dysfunction (EED), also known as environmental enteropathy, is an under-diagnosed condition that is highly prevalent in resource limited settings and known to be implicated in poor growth of young children (4-6). EED is characterized by intestinal inflammation, impaired barrier function and altered intestinal permeability (7). Therefore, several candidate biomarkers including AAT and Reg1B has been proposed and tested as surrogate markers of EED in several studies. However, the exact etiology as well as the pathophysiology of EED is elusive. Prior studies revealed that enteric infections can be a potential cause of altered gut function including inflammatory changes of the gut mucosa and increased intestinal permeability (8). But studies investigating the role of H. pylori infection in alteration of gut health or exacerbation EED have not been carried out previously. Given the high prevalence of both H. pylori infection as well as EED in this patient population, investigation into how each condition influences the other and patient outcomes is of high importance. Therefore, we sought to investigate the association of H. pylori infection with fecal biomarkers of EED and indicators of growth in children hailing from a resource-poor urban settlement in Dhaka, Bangladesh. We have now precisely discussed the rationale of the study in the introduction (page 4, 5).

Study design is appropriate but the samples size calculation has not been given. It is not mentioned if recruitment of 319 children was a convenient sample or based on any calculation.

**Response:** Thank you for your positive comment on study design. The current analysis was done using the dataset of the ongoing Bangladesh Environmental Enteric Dysfunction (BEED) study. We selected 319 children who completed the nutrition intervention, and had data in both the time points. We have mentioned this in the method section under “study design, site and population” (page 6).

Since, it is secondary analysis of data, details of study population are not needed, though some details have been provided here.

**Response:** Thank you for the comment. However, this is not a secondary analysis. We have used the data from the ongoing Bangladesh Environmental Enteric Dysfunction (BEED) study. The objectives of the BEED study include to examine the biology of EED measured in non-invasive candidate biomarkers and to investigate the role of EED in malnutrition. We used the primary data and the aims of this analysis correspond to the objectives of the BEED study. Therefore, we have discussed the study population in detail in the manuscript.

Reviewer #3: A clear hypothesis was not stated: the authors' implied that they sought to investigate the factors associated with H. pylori infection and identify its relationship with the fecal biomarkers of EED. This should be stated clearly.

**Response:** Thank you for the suggestion. The hypothesis has now been discussed in the introduction of the manuscript.

In general, the study was conducted in a straight-forward manner, appropriate methodologies were used to determine H. pylori infection rates and assess biomarkers, and all statistical analysis appears to have been conducted in a thorough and rigorous manner.

**Response:** Thank you very much for the comment.

No concerns exist about ethical / regulatory requirements. The research protocol of this study (protocol no.: PR-16007) was approved by the Institutional Review Board of the International Center for Diarrheal Disease Research, Bangladesh and written informed consent was obtained from the parents or legal guardians.

**Response:** Thank you very much for the comment.

The patient population appears to exhibit slightly lower baseline infection rate of H. pylori when compared to reporting by other groups examining infection rates in Bangladesh.

**Response:** We also agree that the reporting infection rate is lower compared to previous studies done in Bangladesh. Perhaps the use of different methods for detection of H. pylori can be a cause of differences in prevalence between the studies. We have measured the prevalence in children below two years of age. This may not be the sufficient period for acquisition of the infection; hence, the infection rate was low compared to the prior reports. Moreover, the improvement of water, sanitation and hygiene in the community over the past couple of years can be a possible cause for reduction of H. pylori infection in the participants. We have stated this in the discussion section of the manuscript (page 20).

The authors note examples of data appearing to trend in a manner that would support their (likely) preferred conclusion (MPO, Calprotectin, correlation of infection/persistence with stunting). A larger sample size could potentially be one variable that would move these borderline results into the 'significant' category.

**Response:** We also agree with you that a well-designed prospective study with a larger sample size would move the results into the significant category.

--------------------

*<b>Results</b></br></br>*

*-Does the analysis presented match the analysis plan?</br>*

*-Are the results clearly and completely presented?</br>*

*-Are the figures (Tables, Images) of sufficient quality for clarity?</br></br>*

Reviewer #1: The results follow the analysis plan and are clearly described. Study results confirm the acquisition and persistence of H. pylori infection in early childhood in the children living in an urban settlement in Dhaka, Bangladesh. Children living in crowded households were more likely to be affected by the infection with H. pylori. The children with H. pylori infection had higher concentrations of AAT, and lower concentrations of Reg1B in their stool samples.

**Response:** Thank you very much for the positive comment.

Reviewer #2: Though analysis of data is appropriate but table 3 needs to be redesigned. In column of variable, instead of writing H. pylori repeatedly, the markers should have been written. Hence, the table should be redrawn, writing biomarkers in first column and avoiding "Association of Helicobacter pylori infection with ------" in each line.

**Response:** Thank you for the comment. We have now redesigned the table as per your suggestion.

Reviewer #3: The results are clearly and accurately presented.

**Response:** Thank you for the positive comment.

--------------------

*<b>Conclusions</b></br></br>*

*-Are the conclusions supported by the data presented?</br>*

*-Are the limitations of analysis clearly described?</br>*

*-Do the authors discuss how these data can be helpful to advance our understanding of the topic under study?</br>*

*-Is public health relevance addressed?</br></br>*

Reviewer #1: The authors note that around 20% of their study participants, irrespective of their nutritional status, were infected at enrolment. They conclude that H. pylori infection might have an adverse impact on the intestinal health and function of young children in an urban developing country setting. They also point out that further work is required to elucidate the interaction between H. pylori infection and fecal Reg1B concentrations in children during their early years of life.

**Response:** Thank you very much for the comment.

Reviewer #2: Though conclusions are supported by the data but are meaningless. The authors have not discussed at all, how this data can be used to advance understanding of the topic. Just finding of presence or absence of relationship between H. pylori infection and fecal biomarkers of EED doesn't mean anything. Similarly, risk factors for H. pylori infections are well known and nothing new has been found in this study.

**Response:** Thank you for the suggestion. We have now discussed in detail on how the results of this study can be used to advance understanding of the topic. We observed a statistically significant association between H. pylori infection and fecal biomarkers of EED, for instance, AAT and Reg1B through rigorous statistical methods using generalized estimating equations. This is a new finding, and would help better understand the etiology and pathophysiology of EED in young children living in resource poor settings.

Reviewer #3: The conclusions of the study and their impact on public health relevance are vague and understated. They should be more clearly presented in the abstract and discussion sections.

**Response:** The abstract, conclusions, and discussion section have been revised as per your suggestion.

The authors do address some of the limitations inherent to their study design.

**Response:** Thank you for the comment.

--------------------

*<b>Editorial and Data Presentation Modifications?</b></br><br/>*

*Use this section for editorial suggestions as well as relatively minor modifications of existing data that would enhance clarity. If the only modifications needed are minor and/or editorial, you may wish to recommend “Minor Revision” or “Accept”.*

Reviewer #1: This is a well-written paper and I have no suggestions for editorial changes.

Response: Thank you very much.

Reviewer #2: None.

Response: Thank you.

Reviewer #3: (No Response)

Response: Thank you.

--------------------

*<b>Summary and General Comments</b></br></br>*

*Use this section to provide overall comments, discuss strengths/weaknesses of the study, novelty, significance, general execution and scholarship. You may also include additional comments for the author, including concerns about dual publication, research ethics, or publication ethics. If requesting major revision, please articulate the new experiments that are needed.*

Reviewer #1: This study provides a comprehensive approach to understand the interactions between H. pylori infection and consequent gut function during early childhood. The adverse impact of H. pylori infection on childhood growth and development well reported but this study showed insignificant relationship between H. pylori infection and the indicators of nutritional status in the children. The authors comment on the paucity of research investigating the relationship between H. pylori infection and fecal concentrations of AAT. Authors may be interested in the finding from the reviewer in The Gambia (Sullivan PB, Thomas JE, Eastham EJ, Lunn PG, Neale G. Helicobacter pylori and protein losing enteropathy. Archives of disease in childhood. 1990;65(3):332-3.) which found no association between H. pylori and fecal AAT levels in malnourished children with persistent diarrhoea.

**Response:** Thank you for the comment. We have discussed the finding from the reviewer in the discussion section.

Reviewer #2: The manuscript is based on a secondary analysis of data obtained during study on environmental enteric dysfunction, which is already a poorly understood condition. Writing of this manuscript is a share wastage of time, effort and money. This newly described condition, previously known by various names such as tropical enteropathy, etc is not yet an established single disease, nor its diagnostic criteria are well defined. Various blood and stool markers are being investigated, some of those are used in this study and manuscript. In addition, H. pylori has been given an undue importance, role of which has yet to be defined in causation of the EED. Looking for a relationship between H. pylori and fecal markers of EED without any sound hypothesis or reason is not going to lead anywhere. H. pylori infect gastric mucosa, whereas EED is a disease of small intestine. Hence, a strong reason needs to be given to look at the relationship of H. pylori with fecal markers of EED, which has not been given. Even if some relationship is found between H. pylori infection and few markers of EED, it doesn’t explain any cause or effect. Hence, the manuscript is not fit for publication and may be rejected.

**Response:** This manuscript is not based on secondary data. We have used the primary data of the ongoing study entitled Bangladesh Environmental Enteric Dysfunction (BEED) Study (protocol no.: PR-16007). EED is an under-diagnosed condition that is highly prevalent in resource limited settings and known to be implicated in poor growth of young children (4, 9-11). The condition has been described in the scientific literature since 1960s (5). There is no definite criteria for diagnosing EED, but several biomarkers have been tested as markers of EED and found to be associated with features of EED in previous studies (12-14). EED is characterized by persistent immune activation, gut inflammation and altered intestinal permeability resulting from chronic exposure to intestinal pathogens and frequent enteric infections (8, 9, 15). H. pylori is the most frequent enteric infection in developing countries with an estimated prevalence of more than 50% (16). Infection with H. pylori may predisposes to multiple enteric pathogens resulting is altered intestinal health and function (17, 18). But till date, no study investigating the role of H. pylori infection in contributing to altered intestinal health or in exacerbating the features of EED has been carried out in children. Given the high prevalence of both the H. pylori infection and EED in this patient population, investigation into how each condition influences the other is of high importance to this research field.

In addition to above comments, there are several other points which need to be taken into consideration and are discussed below.

1. Stunted and at risk for stunting: I am not sure why the term "at risk for stunting" has been used in this manuscript. Why the authors have labelled children with normal height as "at risk for stunting"? Specially when already a nutrition intervention program is going on. Thus, the children should be labeled as "stunted" and "not stunted".

**Response:** Thank you for the suggestion. In this study, we enrolled children in two groups: stunted [length-for-age z score (LAZ) <2] and at risk of stunting [LAZ = −1 to −2]. The children who were at risk of being stunted had a marginal LAZ score [LAZ = −1 to −2]; and therefore, we have termed them as “at risk of stunting”. We also mentioned the term in our published protocol paper (19). Hence, we think that it would be better to use “at risk of stunting” instead of “non-stunted” to maintain the consistency as well as to demonstrate the exact nutritional status of the children of this group.

2. Regarding H. pylori infection, it is mentioned that one-fifth of the participants had H. pylori infection at both the time points, with 13.8% overall persistence. These children should have been divided into three groups, a) Those who had infection at recruitment but got cleared by the end of study. b) those children who remained infected and c) who acquired new infection during the study.

These three groups should be analyzed separately and compared. This probably could show if children with abnormal fecal biomarkers are prone to H. pylori infection or H. pylori infection led to abnormal fecal biomarkers and EED.

**Response:** We have divided the children into four groups as per your suggestion. The groups are: a) those who had infection at recruitment but got cleared by the end of study, b) those children who remained infected, c) who acquired new infection during the study, and d) who remained non-infected in both the time points. We consider this as a new variable with four categories. Subsequently, multivariable linear regression was applied considering the biomarker values at the end of nutrition intervention as the outcome variables. All the biomarker values were log-transformed prior to analysis. We considered the children who remained non-infected as the reference group. Please find below the result of the analysis.

| **Variables** | **AAT,** mg/g | **MPO,** ng/mL | **NEO,** nmol/L | **Calprotectin,** μg/g | **Reg1B,** μg/mL |
| --- | --- | --- | --- | --- | --- |
| Age in days | -0.03 (-0.09, 0.03) | -0.04 (-0.08, 0.01) | -0.08 (-0.14, -0.02) * | -0.05 (-0.11, 0.01) | -0.11 (-0.19, -0.03) * |
| Sex (female) | -0.11(-0.31, 0.09) | -0.003 (-0.20, 0.19) | 0.16 (-0.04, 0.36) | -0.12 (-0.36, 0.12) | 0.08 (-0.23, 0.39) |
| Nutritional status  (At risk of stunting) | -0.19(-0.39, 0.01) | 0.05(-0.15, 0.25) | -0.08 (-0.28, 0.12) | 0.03 (-0.21, 0.27) | -0.06 (-0.39, 0.27) |
| *Helicobacter pylori* infection status  (Ref: non-infected) |  |  |  |  |  |
| Infected at enrolment | 0.58(0.05, 1.11) * | 0.67 (0.16, 1.18) * | 0.19 (-0.32, 0.70) | 0.73 (0.10, 1.36) * | 0.11 (-0.73, 0.95) |
| Infected acquired during study | 0.33(-0.04, 0.70) | 0.30(-0.07, 0.67) | -0.72 (-1.09, -0.35) * | 0.29 (-0.14, 0.72) | -0.68 (-1.27, -0.10) * |
| Remained infected | -0.09(-0.38, 0.20) | 0.14(-0.15, 0.43) | -0.09 (-0.38, 0.20) | 0.27 (-0.08, 0.62) | -0.67 (-1.14, -0.20) * |

*Each column represents an individual model. Biomarker values were log-transformed prior to analysis. Coefficient values with standard errors have been reported in the table. The asterisk (*) sign indicates the statistical significance.*

We observed that children who were infected at enrolment had significantly higher fecal concentrations of AAT (p-value = 0.03), MPO (p-value = 0.01), and Calprotectin (p-value = 0.02) at the end of nutrition intervention compared to the children who remained non-infected. Children who acquired infection during the study (p-value = 0.03) and who remained infected in both the time points (p-value = 0.006) had significantly lower concentrations of fecal Reg1B compared to the reference group. Fecal NEO concentration was significantly lower (p-value < 0.001) in children who acquired infection during the study in comparison to the children who had no infection at all. The findings of this linear regression analyses support the role of H. pylori in intestinal inflammation as well as increased gut permeability, the two important domains of EED. This result has now been included in the manuscript (page 17).

3. To look at any association between H. pylori infection and fecal biomarkers, normal children without EED should have been included in the study. Only then, one would be able to determine if H. pylori infection has any relationship with these fecal biomarkers. If any such association is found in normal children i.e. if normal children without EED but infected with H. pylori are found to have these fecal biomarkers in stool, it may be inferred that H. pylori may have a role in causation of EED. If not so then EED might predispose children to acquire H. pylori infection. Presence or absence of relationship between H. pylori and fecal biomarkers without looking at the pathogenesis or cause and effect doesn't mean anything, particularly when H. pylori group is a heterogenous group.

**Response:** We also agree with you it would be better if we could we include healthy children without EED as control group. It has already been mentioned as a limitation of the study. We determined the association between infection with H. pylori and fecal biomarkers of EED using multivariable linear regression with generalized estimating equations (GEE). GEE is a robust statistical method for analyzing correlated data and provides unbiased estimation of regression coefficients (20). Therefore, we can confirm that the association determined in this study using GEE analysis is statistically appropriate and provides insight for further evaluation of our study objectives.

4. Tables should be drawn in standard way without any grid line.

**Response:** Tables has been redesigned as per your suggestion.

5. Table 3 is poorly drawn. In column of variable, instead of writing H. pylori repeatedly, the markers should have been written. Hence, the table should be redrawn, writing biomarkers in first column and avoiding "Association of Helicobacter pylori infection with ------" in each line.

**Response:** Table 3 has been restructured as per your suggestion.

Reviewer #3: The authors sought to investigate the factors associated with H. pylori infection and identify its relationship with the fecal biomarkers of EED including Myeloperoxidase (MPO), Neopterin (NEO), Calprotectin, Reg1B and Alpha-1 antitrypsin (AAT), and nutritional status of the children in their patient cohort in Bangladesh. The study found a positive association with AAT, an inverse relationship with Reg1B, and no association with the other biomarkers examined, nutritional state and/or stunting in children. The authors state that their findings affirmed the acquisition and persistence of H. pylori infection, which they claimed might account for altered gut function in the early years of life.

**Response:** Thank you for the comment.

The overall concept defining the study is interesting and novel: a correlative study investigating H. pylori infections with the potential to exacerbate EED has not been carried out previously and given the high prevalence of both in this patient population, investigation into how each condition influences the other and patient outcomes is of high importance to the field.

**Response:** Thank you very much for your very positive response.

In general, the study was conducted in a straight-forward manner, appropriate methodologies were used to determine H. pylori infection rates and assess biomarkers, and all statistical analysis appears to have been conducted in a thorough and rigorous manner. The manuscript is well organized and written clearly enough to be accessible to non-specialists. The only major issue with the manuscript in its current form is that the conclusions that the authors draw from the data are somewhat hard to interpret. This may be due to the relative ‘mixed bag’ of their reported results, however, the authors need to try and address these potential discrepancies in the context of their hypothetical model. In this light, the project’s overall goals and overarching hypothesis need to be more clearly stated in the abstract, discussion, and conclusion sections. I have provided some ideas on how the authors might wish to address this below:

**Response:** Thank you for the suggestions. We have revised the abstract, discussion, and conclusion sections following your suggestions.

Major comments:

The major limitation of this study is its lack of a clearly-defined, testable research objective, and the consequent weakness of the authors’ overall conclusions. ‘Hypothesis’ was not mentioned once in the manuscript, but if one was to be inferred from the author’s introduction, it would be that H. pylori infections contribute to and/or exacerbate Environmental Enteric Dysfunction (EED) and subsequent growth failure in children. In their conclusions, rather than state whether this hypothesis was supported (or not) by the data generated in this study, the author’s hedge and conclude what is already well-known to the field; that the acquisition and persistence of H. pylori infection occurs early in life. If this is the only definitive conclusion of this study, then it is not of sufficient novelty for publication in PLOS Neglected Tropical Diseases … because we already know this. The novelty comes from whether or not H. pylori infection exacerbates EED and the authors need to do 1 of 3 things to address this potential hypothesis:

1) State that infection does correlate with an exacerbated EED status based on the AAT / Reg1B data and offer a convincing explanation (preferably backed up with either experimentation or the citation of the experimentation of others) as to why AAT (a readout of enteric protein loss / intestinal permeability) and Reg1B (tissue injury/repair) might be better indicators of EED status in children than MPO, NEO, and Calprotectin (which offer readouts of inflammation)

2) State that exacerbated EED status should result in elevated levels for all biomarkers examined (AAT, MPO, NEO, and Calprotectin) and thus conclude that H. pylori infection does not appear to exacerbate EED in this patient cohort

3) State that the data was inconclusive and suggest reasons for this (potentially due to inadequate sampling size, robustness of the assay, biomarkers of EED failing to capture a perfect picture of disease abundance in general, etc.)

Given that EED has been previously shown to affect MPO, NEO, Calprotectin, child growth/development/nutritional status and that none of these appear to be different between Hp infected and non-infected individuals in this study, the data would appear to favor #2.

**Response:** Thank you for the suggestions. We have revised the manuscript and stated the hypothesis of this study in the introduction of the revised draft. The conclusions have been edited according to the hypothesis of the study. EED is an inconclusive entity and the definite diagnostic criteria is yet to be confirmed. However, several studies considered the measures of gut health, for instance, AAT, MPO, NEO as the potential candidates to evaluate EED. Fecal concentrations of AAT can be a better candidate to EED. AAT is a protein that is released by neutrophils during infection or inflammatory conditions (11, 21). Since AAT is not produced in the intestine, its presence in the gut lumen can be considered as a measure of gut inflammation (11). AAT also reflects protein loss due to disruption of mucosal barrier and has been established as a marker of intestinal permeability (11, 22). Because AAT can be used for assessing both intestinal inflammation and increased gut permeability – two important domains of EED – several studies measured fecal AAT as a biomarker of EED (5, 6, 23, 24). Prior report showed that fecal AAT as well as “EED composite score” comprising AAT were associated with impaired linear growth in children (5, 25). Therefore, we assume that AAT measured in the stool samples can be a better readout for diagnosing EED. We have now included this explanation in the discussion of the manuscript (page 20, 21).

It is curious that MPO, Calprotectin, and NEO (readouts for intestinal inflammation) were not significantly different between infected and non-infected individuals, and yet both AAT (associated with protein loss / intestinal permeability) and Reg1B (associated with epithelial tissue injury and subsequent repair) were significantly different. Regardless of their eventual decision on the overall conclusions of this study, the authors should present an explanation for why epithelial tissue injury and intestinal protein loss (presumably from an active infection) might not result in subsequent inflammation (which is generally highly abundant during Hp infections, although presumably less so in the small intestine).

**Response:** We also wonder why epithelial tissue injury, increased gut permeability and intestinal protein loss did not result in subsequent inflammation in children of this cohort. Perhaps, follow up for a longer period would help us to better understand the phenomenon. We assume that the first two years of life is the time for acquisition of the infection, and it requires more time from H. pylori infection to intestinal inflammation mediated by intestinal damage, repair and increased intestinal permeability. However, we did another analysis following the suggestion of one of our reviewers that has now been included in the manuscript. We created a new categorical variable based on the H. pylori status at enrollment and at the end of nutrition intervention. The four categories of the new variable are: a) those who had infection at recruitment but got cleared by the end of study, b) those children who remained infected, c) who acquired new infection during the study, and d) who remained non-infected in both the time points. We then performed multivariable linear regression analysis considering the fecal biomarker values at the end of nutrition intervention as the outcome variables. The results showed that children who had H. pylori infection at enrollment but cleared at the end of the study had significantly higher fecal concentrations of AAT, MPO and calprotectin. This finding suggests that the infection with H. pylori have substantial impact on gut health and the impact may persist for a considerable period of time even after resolution of the infection.

Line 42-43: Consider modification to: “Infection was not associated with nutritional status of the children, nor was it associated with indicators of childhood growth.”

**Response:** The sentence has been modified as per your suggestion.

Lines 68-89: Isenbarger et al (1998) found that the incidence of diarrhea was not significantly different between children by H. pylori serostatus and concluded that H. pylori infection was not associated with an increased risk of diarrheal disease. Quinonez et al (1999) determined that Helicobacter pylori appeared to have no effect on the nutritional status of the studied children and that differences detected were small and likely due to sociodemographic factors. Vilchis et al (2009) was the only one of the three studies cited to suggest that H. pylori infection has a negative effect on the growth of children. A better citation would be Franceschi et al 2014, World Journal of Gastroenterology (and the articles cited therein) as they summarize a number of studies that have found positive associations with H. pylori incidence and malnutrition as well as explore mechanistic aspects of H. pylori-caused malnutrition. Overall (as the authors imply in lines 76-77 and later on in the discussion section) the data on the topic reported to date are decidedly a mixed bag. The introduction should also make this clear.

**Response:** Thank you very much for the suggestion. We have revised the introduction and cited the articles you have referred in your comment.

Figure 1: As the authors note, the number of H. pylori infected individuals in the author’s dataset appears to be on the low side for Bangladesh, with only 20% of the participants testing positive for H. pylori. Rates of infection of up to 60% have been reported in the population at large (Habib 2016) and in infants aged 1-3 months (Mahalanabis 1996, who also reported finding no association with H. pylori infection and nutritional state). The author’s should comment on how this might affect the interpretation of their results.

**Response:** We agree that the prevalence estimated in this study is lower compared to previous reports. Perhaps the use of different methods for detection of H. pylori can be a cause of differences in prevalence between the studies. We have measured the prevalence in children below two years of age. This may not be the sufficient period for acquisition of the infection, and hence, the infection rate was low compared to the prior reports. Moreover, the improvement of water, sanitation and hygiene in the community over the past couple of years can be attributable to the reduction of H. pylori infection in the study population. We have discussed in detail in the discussion on how this lower prevalence might affect the relationship between H. pylori and nutritional status of the young children.

Line 215-216: " ... all the fecal biomarker values were reduced significantly after the 90-day nutrition intervention (p-value <0.05)." This would seem to be a rather significant finding: do the authors take this as evidence of successfully combating EED with nutritional intervention? If so, they should state as much.

**Response:** The significant reduction of fecal biomarker values after the 90-day nutrition intervention indicates the improvement of gut health among the children. Since our aim was not to investigate the effect of nutrition intervention in this analysis, we did not test the effect of diets in successfully combating EED. The result is striking, but should be investigated more rigorously to reach an inference. However, we have discussed the result in discussion.

Line 259-260: This data is in line with previous reports (most recently Aitila 2019) showing higher prevalence in crowded households.

**Response:** Thank you for the reference. We have cited the article in discussion.

--------------------

*PLOS authors have the option to publish the peer review history of their article (<a href="https://journals.plos.org/plosntds/s/editorial-and-peer-review-process#loc-peer-review-history" target="_blank">what does this mean?</a>). If published, this will include your full peer review and any attached files.<br><br>*

*If you choose “no”, your identity will remain anonymous but your review may still be made public.<br><br>*

*<b>Do you want your identity to be public for this peer review?</b> For information about this choice, including consent withdrawal, please see our <a href="https://www.plos.org/privacy-policy" target="_blank">Privacy Policy</a>.*

Reviewer #1: Yes: Peter B Sullivan

Reviewer #2: No

Reviewer #3: No

**References:**

1.	Aitila P, Mutyaba M, Okeny S, Ndawula Kasule M, Kasule R, Ssedyabane F, et al. Prevalence and Risk Factors of Helicobacter pylori Infection among Children Aged 1 to 15 Years at Holy Innocents Children’s Hospital, Mbarara, South Western Uganda. Journal of tropical medicine. 2019;2019.

2.	Hill ID, Sinclair-Smith C, Lastovica A, Bowie M, Emms M. Transient protein losing enteropathy associated with acute gastritis and campylobacter pylori. Archives of disease in childhood. 1987;62(12):1215-9.

3.	Landzberg BR, Pochapin MB. Protein-losing enteropathy and gastropathy. Current treatment options in gastroenterology. 2001;4(1):39-49.

4.	Liu T-C, VanBuskirk K, Ali SA, Kelly MP, Holtz LR, Yilmaz OH, et al. A novel histological index for evaluation of environmental enteric dysfunction identifies geographic-specific features of enteropathy among children with suboptimal growth. PLOS Neglected Tropical Diseases. 2020;14(1):e0007975.

5.	Kosek M, Haque R, Lima A, Babji S, Shrestha S, Qureshi S, et al. Fecal markers of intestinal inflammation and permeability associated with the subsequent acquisition of linear growth deficits in infants. The American journal of tropical medicine and hygiene. 2013;88(2):390-6.

6.	Arndt MB, Richardson BA, Ahmed T, Mahfuz M, Haque R, John-Stewart GC, et al. Fecal markers of environmental enteropathy and subsequent growth in Bangladeshi children. The American journal of tropical medicine and hygiene. 2016;95(3):694-701.

7.	Fahim SM, Das S, Sanin KI, Gazi MA, Mahfuz M, Islam MM, et al. Association of Fecal Markers of Environmental Enteric Dysfunction with Zinc and Iron Status among Children at First Two Years of Life in Bangladesh. The American journal of tropical medicine and hygiene. 2018;99(2):489-94.

8.	Fahim SM, Das S, Gazi MA, Mahfuz M, Ahmed T. Association of intestinal pathogens with faecal markers of environmental enteric dysfunction among slum‐dwelling children in the first 2 years of life in Bangladesh. Tropical Medicine & International Health. 2018;23(11):1242-50.

9.	Kosek MN, Ahmed T, Bhutta Z, Caulfield L, Guerrant R, Houpt E, et al. Causal pathways from enteropathogens to environmental enteropathy: findings from the MAL-ED birth cohort study. EBioMedicine. 2017;18:109-17.

10.	Kelly P, Besa E, Zyambo K, Louis-Auguste J, Lees J, Banda T, et al. Endomicroscopic and transcriptomic analysis of impaired barrier function and malabsorption in environmental enteropathy. PLoS neglected tropical diseases. 2016;10(4).

11.	Harper KM, Mutasa M, Prendergast AJ, Humphrey J, Manges AR. Environmental enteric dysfunction pathways and child stunting: A systematic review. PLoS neglected tropical diseases. 2018;12(1):e0006205.

12.	Uddin MI, Islam S, Nishat NS, Hossain M, Rafique TA, Rashu R, et al. Biomarkers of environmental enteropathy are positively associated with immune responses to an oral cholera vaccine in Bangladeshi children. PLoS neglected tropical diseases. 2016;10(11).

13.	McCormick BJ, Lee GO, Seidman JC, Haque R, Mondal D, Quetz J, et al. Dynamics and trends in fecal biomarkers of gut function in children from 1–24 months in the MAL-ED study. The American journal of tropical medicine and hygiene. 2017;96(2):465-72.

14.	George CM, Oldja L, Biswas S, Perin J, Lee GO, Kosek M, et al. Geophagy is associated with environmental enteropathy and stunting in children in rural Bangladesh. The American journal of tropical medicine and hygiene. 2015;92(6):1117-24.

15.	George CM, Burrowes V, Perin J, Oldja L, Biswas S, Sack D, et al. Enteric infections in young children are associated with environmental enteropathy and impaired growth. Tropical Medicine & International Health. 2018;23(1):26-33.

16.	Ortiz-Princz D, Daoud G, Salgado-Sabel A, Cavazza M. Helicobacter pylori infection in children: should it be carefully assessed. Eur Rev Med Pharmacol Sci. 2016;20(9):1798-813.

17.	Salama RI, Emara MH, Mostafa HM, Abd-Elsalam S, Alnabawy SM, Elshweikh SA, et al. Helicobacter pylori infection and risk of salmonella infection. Medicine. 2019;98(6).

18.	Franceschi F, Annalisa T, Di Rienzo Teresa D, Ianiro G, Franco S, Viviana G, et al. Role of Helicobacter pylori infection on nutrition and metabolism. World Journal of Gastroenterology: WJG. 2014;20(36):12809.

19.	Mahfuz M, Das S, Mazumder RN, Rahman MM, Haque R, Bhuiyan MMR, et al. Bangladesh environmental enteric dysfunction (BEED) study: protocol for a community-based intervention study to validate non-invasive biomarkers of environmental enteric dysfunction. BMJ open. 2017;7(8):e017768.

20.	Ghisletta P, Spini D. An introduction to generalized estimating equations and an application to assess selectivity effects in a longitudinal study on very old individuals. Journal of Educational and Behavioral Statistics. 2004;29(4):421-37.

21.	De Serres F, Blanco I. Role of alpha‐1 antitrypsin in human health and disease. Journal of internal medicine. 2014;276(4):311-35.

22.	Fahim SM, Das S, Sanin KI, Gazi MA, Mahfuz M, Islam MM, et al. Association of Fecal Markers of Environmental Enteric Dysfunction with Zinc and Iron Status among Children at First Two Years of Life in Bangladesh. The American journal of tropical medicine and hygiene. 2018.

23.	Campbell RK, Schulze K, Shaikh S, Mehra S, Ali H, Wu L, et al. Biomarkers of environmental enteric dysfunction among children in rural Bangladesh. Journal of pediatric gastroenterology and nutrition. 2017;65(1):40.

24.	George CM, Oldja L, Biswas SK, Perin J, Lee GO, Ahmed S, et al. Fecal markers of environmental enteropathy are associated with animal exposure and caregiver hygiene in Bangladesh. The American journal of tropical medicine and hygiene. 2015;93(2):269-75.

25.	Lima AA, Leite AM, Moura AF, Lima NL, Oria RB, Soares AM, et al., editors. Risk factors, gut function biomarkers and growth deficit associated with environmental enteropathy and malnutrition: The case-control mal-ed study in Fortaleza, Ceara, Brazil. American Journal of Tropical Medicine and Hygiene; 2015: AMER SOC TROP MED & HYGIENE 8000 WESTPARK DR, STE 130, MCLEAN, VA 22101 USA.
